# Experimental maturation of Archaea encrusted by Fe-phosphates

**DOI:** 10.1038/s41598-017-17111-9

**Published:** 2017-12-05

**Authors:** J. Miot, S. Bernard, M. Bourreau, F. Guyot, A. Kish

**Affiliations:** 1 IMPMC, Sorbonne Université, MNHN, UPMC, CNRS UMR 7590, 4 pl. Jussieu, 75005 Paris, France; 2 MCAM, MNHN, UPMC, CNRS UMR 7245, 63 rue Buffon, 75005 Paris, France

## Abstract

Burial is generally detrimental to the preservation of biological signals. It has often been assumed that (bio)mineral-encrusted microorganisms are more resistant to burial-induced degradation than non-encrusted ones over geological timescales. For the present study, we submitted *Sulfolobus acidocaldarius* experimentally encrusted by amorphous Fe phosphates to constrained temperature conditions (150 °C) under pressure for 1 to 5 days, thereby simulating burial-induced processes. We document the molecular and mineralogical evolution of these assemblages down to the sub-micrometer scale using X-ray diffraction, scanning and transmission electron microscopies and synchrotron-based X-ray absorption near edge structure spectroscopy at the carbon K-edge. The present results demonstrate that the presence of Fe-phosphates enhances the chemical degradation of microbial organic matter. While Fe-phosphates remained amorphous in abiotic controls, crystalline lipscombite (Fe^II^_x_Fe^III^_3−x_(PO_4_)_2_(OH)_3−x_) entrapping organic matter formed in the presence of *S. acidocaldarius* cells. Lipscombite textures (framboidal vs. bipyramidal) appeared only controlled by the initial level of encrustation of the cells, suggesting that the initial organic matter to mineral ratio influences the competition between nucleation and crystal growth. Altogether these results highlight the important interplay between minerals and organic matter during fossilization, which should be taken into account when interpreting the fossil record.

## Introduction

Reconstructing the evolution of life during geological times requires to properly decode the organic fossil record. Although ancient biomolecules may withstand advanced diagenesis in some contexts^1–4^, burial-induced degradation processes are usually detrimental to microbial morphology and chemistry^5,6^. The critical role of authigenic mineralization (*e.g*., silicification, pyritization, calcification, phosphatization, chloritization, glauconitization) in the morphological and chemical survivability of organic fossils through geological times has been recognized and highlighted by many authors^1,7–13^. In parallel, laboratory experiments have offered the opportunity to evaluate the influence of multiple key parameters on biogenic organic matter evolution upon diagenetic conditions^14–16^. In particular, a few studies have focused on the influence of the nature of the mineral phase by submitting (bio)encrusted bacteria^17–19^, experimentally entombed microorganisms^20,21^ or mixtures of minerals and organic molecules^22,23^ to well constrained pressure and/or temperature conditions, thereby simulating natural fossilization processes in the lab. In addition, this experimental approach allows evaluating the influence of organic matter maturation onto (bio)mineral transformations.

Fe minerals are widespread in the fossil record, especially in the precambrian Banded Iron Formations^24,25^. But an experimental evaluation of their impact on organic matter preservation, of their evolution at the contact of organic matter upon diagenetic conditions and hence of their ability to record biosignatures remains scarce. A diversity of microbial Fe biomineralization pathways have been described leading to a variety of Fe mineral products, including Fe (oxyhydr)oxides^26^, Fe sulfides^27,28^ and Fe phosphates^29,30^. Except photoferrotrophy that leads to spatially segregated organic matter and Fe phases^31^, most of the known Fe biomineralization pathways lead to fine scale associations of Fe minerals with microbial structures^32^. Most archaea and Gram positive and negative bacteria exhibit charged groups exposed at their surface, thereby promoting the adsorption (or the preferential precipitation) of Fe cations and Fe minerals at their contact^33–35^. Microorganisms encrusted by Fe minerals are widespread in modern ferruginous environments^36,37^ and may thus have been commonplace in ancient ferruginous habitats. For instance, Fe-phosphate encrusted cells have been observed in the anoxic layer of the meromictic and ferruginous lake Pavin^38,39^, considered as a possible analogue of a precambrian stratified ocean^40,41^. However, the evolution of cells encrusted by Fe-phosphates upon sedimentation and diagenesis remains unexplored.

Here, we submitted cells of the archaeon *Sulfolobus acidocaldarius* encrusted by Fe-phosphates to diagenetic conditions in closed systems. This strain is an aerobic hyperthermophile isolated from metal-rich acidic solfatares and hot springs^42^. It grows at 55 to 85 °C under acidic conditions (pH 1–6) in the presence of sulfur and various metals including Fe^42^. Of importance, *Sulfolobus acidocaldarius* display, on the outer part of their cell wall, highly ordered crystalline proteinaceous structures (S-layers) that can become encrusted by amorphous Fe-phosphates with fine ultrastructural details being preserved upon encrustation^43^. Here, we compare the evolution of Fe-phosphate-encrusted and non-encrusted *Sulfolobus acidocaldarius* cells exposed to 150 °C for 1 to 5 days. The combination of X-ray diffraction (XRD), scanning and transmission electron microscopies (SEM and TEM) and X-ray absorption near edge structure (XANES) spectroscopy at the C K-edge, allows documenting down to the sub-micrometer scale the morphological, mineralogical and organic geochemical evolution of these organo-mineral assemblages exposed to hydrothermal conditions typical of diagenetic settings.

## Materials and Methods

### Culture and encrustation of *Sulfolobus acidocaldarius*

Cells of *Sulfolobus acidocaldarius* s. DSM 639 were cultured in growth medium (Brock’s medium^42^ pH 3.5 supplemented with 0.1% yeast extract and 0.2% D-saccharose, 170 rpm agitation) then transferred into a mineralization medium^43^. Briefly, cells were cultured under heterotrophic conditions at 80 °C aerobically (resulting in an average generation time of 6 h) until reaching the mid-exponential growth phase (OD_600nm_ = 0.6). Cells were then harvested by centrifugation (8000 *g*, 10 min) and washed twice in MilliQ water. Some of these rinsed cells were resuspended in a mineralization medium composed of 10 mM FeSO_4_ and 10 mM NaH_2_PO_4_ (pH 4.5) at a density of approximately 3.5 10^10^ cells/mL. After 6 h or 24 h of incubation, cells were harvested by centrifugation (8000 *g*, 10 min), then washed twice in MilliQ water. An abiotic control was prepared by mixing 10 mM FeSO_4_ and 10 mM NaH_2_PO_4_ at pH 4.5. Amorphous Fe-phosphates were then collected by centrifugation (8000 *g*, 10 min) and washed twice in MilliQ water.

### Experimental fossilization

Abiotic control, non-encrusted cells and cells encrusted for 6 and 24 h were resuspended in 2 mL of milliQ water and placed within Parr© PTFE reactors maintained at 150 °C ~ 5 bars (saturation vapor pressure) for 1 to 5 days. At the end of the experiments, the suspensions were recovered and stored at 4 °C before analyses.

### X-ray Diffraction (XRD)

The bulk mineralogical compositions of the residues and the starting materials were determined using the Panalytical X’pert Pro X-ray diffractometer operating at IMPMC (Paris, France) at 40 kV and 40 mA (Co Kα radiation). A few mL of suspension of each sample were dried on a silicium sample holder, then analyzed in the 20°–100° 2θ angle range, with a step size of 0.001° (2θ) for a minimum total counting time of 2 h per sample.

### Scanning Electron Microscopy (SEM)

For SEM observations, about 100 µL of suspension of each sample were filtered through a 0.2 µm GTTP polycarbonate filter in a Swinnex filter holder (Merck Millipore, Darmstadt, Germany). Dried filters were carbon coated and analyzed using the Zeiss Ultra 55 SEM operating at IMPMC (Paris, France) and equipped with a field emission gun and a Brucker EDS QUANTAX detector (Brucker Corporation, Houston, TX, USA). Imaging was performed in secondary electron mode (In Lens detector) at 5 kV and a working distance of 3 mm. Energy dispersive X-ray spectrometry (EDXS) analyses and mapping were performed at 15 kV and a working distance of 7.5 mm after calibration with reference copper.

### Focused Ion Beam (FIB) milling

Focused ion beam (FIB) milling was performed using the FEI STRATA DB 235 FIB system operating at the IEMN (Lille, France) to prepare electron transparent 80 nm-thick sections^44,45^ across minerals from the experimental residues. Milling at low Ga-ion currents allowed preventing common artifacts like local gallium implantation, mixing of components, creation of vacancies or interstitials, formation of amorphous layers, local composition changes or redeposition of the sputtered material on the sample surface^46,47^.

### Transmission Electron Microscopy (TEM) sample preparation and analyses

FIB sections were analyzed using scanning transmission electron microscopy (STEM) and high resolution TEM (HRTEM). STEM and HRTEM observations were performed using the 200 kV field emission gun (FEG) JEOL2100F microscope operating at IMPMC (Paris, France). STEM observations were performed in high-angle annular dark field (HAADF) mode. Selected-area electron diffraction (SAED) patterns were obtained on areas of interest. To complete these analyses, starting materials were fixed in 0.1 N sodium cacodylate buffer (pH 7.4) containing 2.5% glutaraldehyde at 4 °C, post-fixed for 1 h via the addition of 1% OsO_4_ and washed three times in 0.1 N sodium cacodylate buffer (pH 7.4), dehydrated in increasing concentrations of ethanol and progressively embedded in Spurr resin. These cells were then cut (70 nm-thick sections) with a Reichert-Yung Ultracut E ultramicrotome and deposited onto 100-mesh formvar coated copper grids. They were observed with a JEOL2100 microscope equipped with a LaB_6_ electron source and operating at 200 kV.

### Scanning Transmission X-ray Microscopy (STXM)

Experimental residues were directly deposited onto formvar copper grids before analysis. STXM analyses were carried out at the HERMES beamline at SOLEIL (Saint Aubin, France)^48^. Energy calibration was accomplished using the well-resolved 3p Rydberg peak at 294.96 eV of gaseous CO_2_ for the C K-edge. XANES data were obtained by collecting image stacks from 270 to 350 eV with increments of 0.1 eV over areas of several µm², with a dwell time of ca. 1 millisecond per pixel in order to avoid irradiation damage, following the procedures for radiation sensitive samples recommended by Wang *et al*.^49^. Alignment of image stacks and extraction of the XANES spectra was done using the aXis2000 software^50^. Following Alléon *et al*.^14^, Barré *et al*.^51^ and Vinogradoff *et al*.^14,51,52^, XANES spectra were normalized to their area between 280 eV and 291.5 eV, thereby ensuring chemical consistency (a spectrum showing a prominent absorption at a given energy must have a less intense absorption at the energy of the other functional groups).

## Results

### Starting Materials

**Figure 1:**
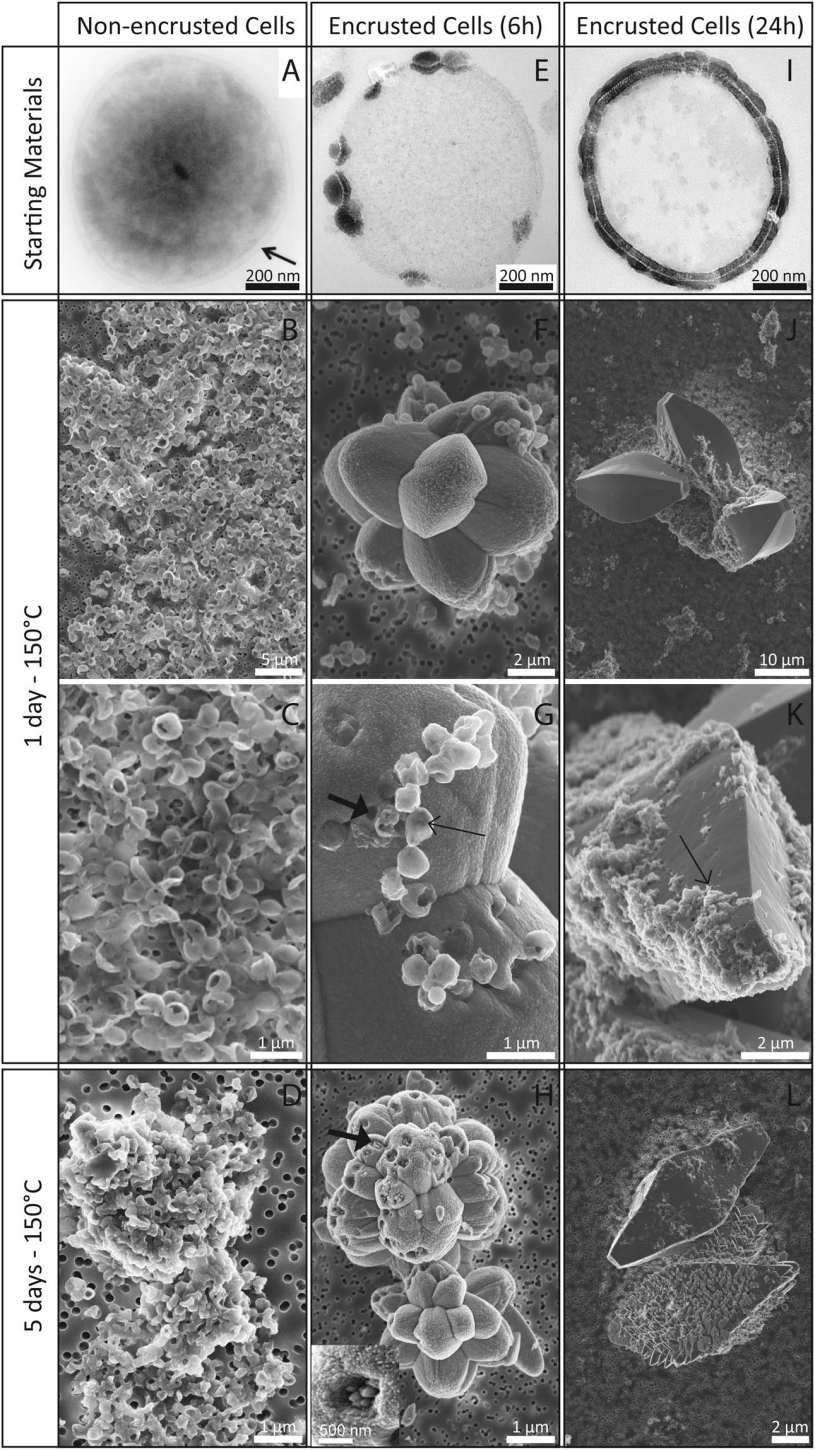
Evolution of non-encrusted and encrusted *S. acidocaldarius* cells upon heating at 150 °C for 1 to 5 days. TEM images of thin sections of the starting materials (before heating, first row) and SEM images of the heated samples. Thin arrows point out non mineralized vesicles (**G**) and patches of organic matter (**K**). Thick arrows point out pores at the surface of the framboids (**G**,**H**). Inset in panel H shows a detail of a partly filled hole.

**Figure 2:**
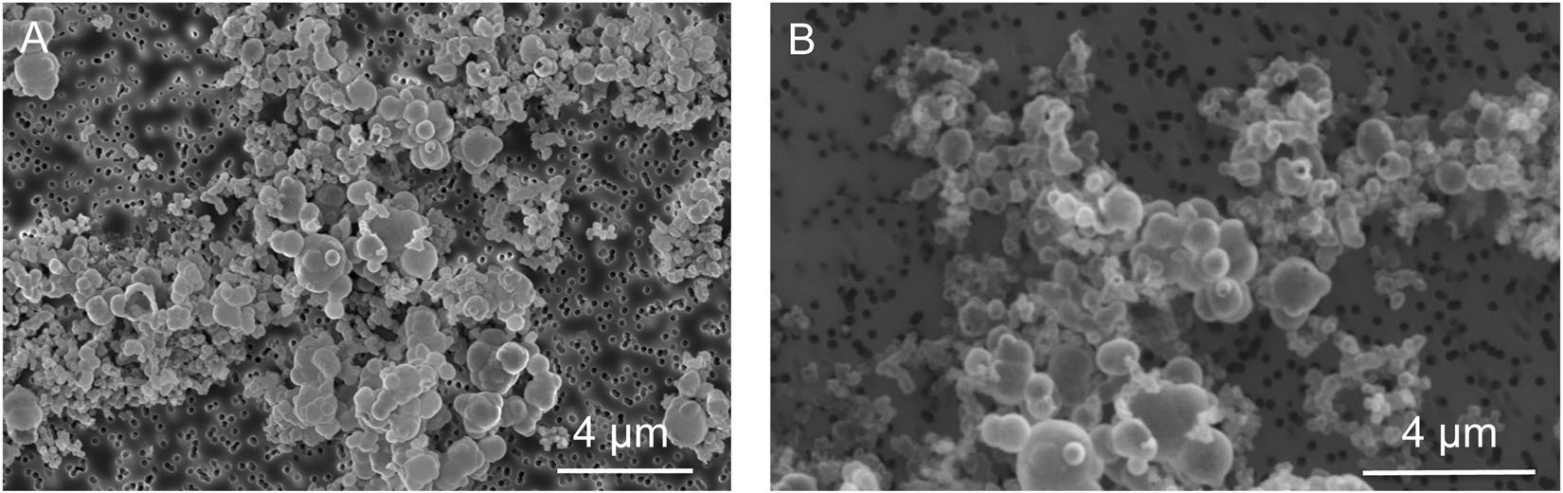
SEM images of abiotic controls (Fe phosphate precipitates) before heating (**A**) and after heating at 150 °C for 5 days (**B**).

**Figure 3:**
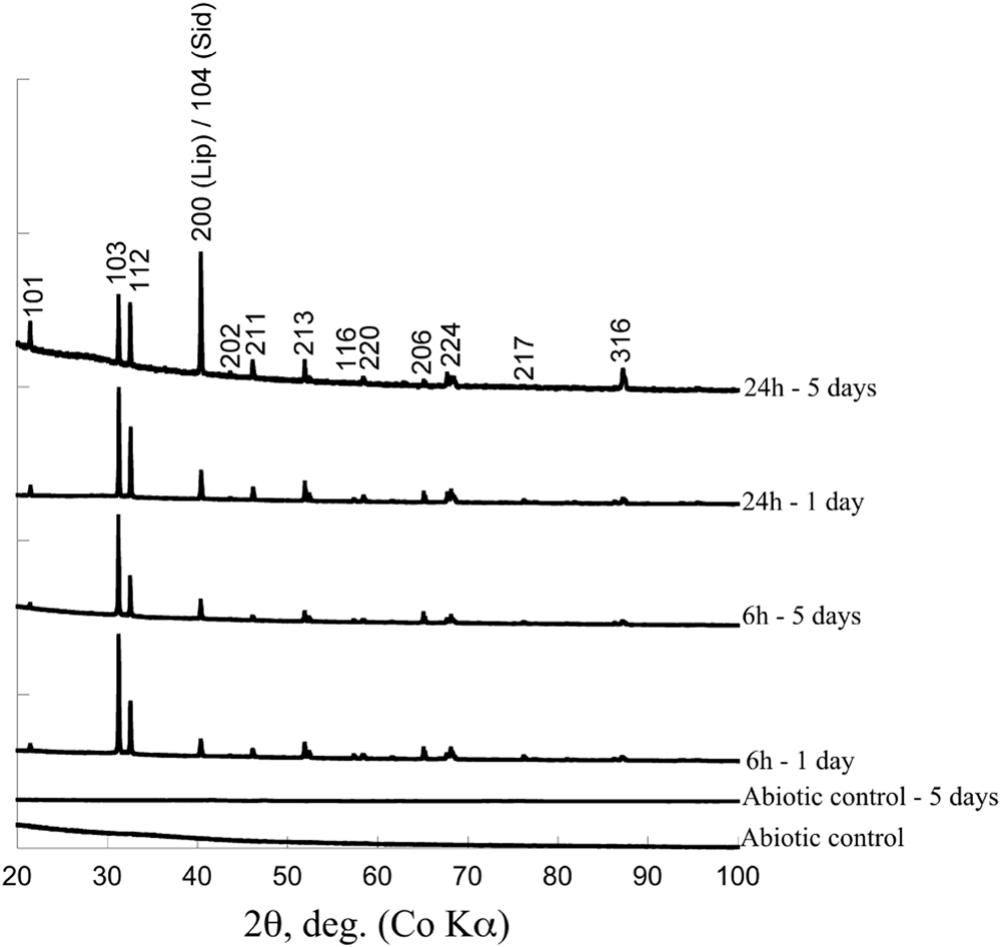
XRD analyses of abiotic controls and *S. acidocaldarius* samples (mineralized for 6 h or 24 h) and heated for 1 day or 5 days in close system. Non labeled *(hkl)* planes are those of lipscombite. The *(200)* plane of lipscombite overlaps with the *(104)* plane of siderite.

Non-encrusted cells of *Sulfolobus acidocaldarius* exhibited spherical morphologies and were composed of a cell membrane surrounded by a proteinaceous S-layer. The cell membrane became encrusted by amorphous Fe-phosphates if immersed in the mineralization medium^43^. After 6 h of incubation in this medium, cells were only partly covered with globules of Fe phosphates evolving towards a continuous layer of Fe phosphates after 24 h (Fig. 1). Abiotic Fe-phosphates that precipitated in the same medium at pH 4.5 consisted of nanometric particles of 200–300 nm in diameter having a composition close to FePO_4_.3(H_2_O)^35^ (Fig. 2). They were amorphous as revealed by XRD (Fig. 3).

### Organic geochemical evolution upon experimental fossilization

**Figure 4:**
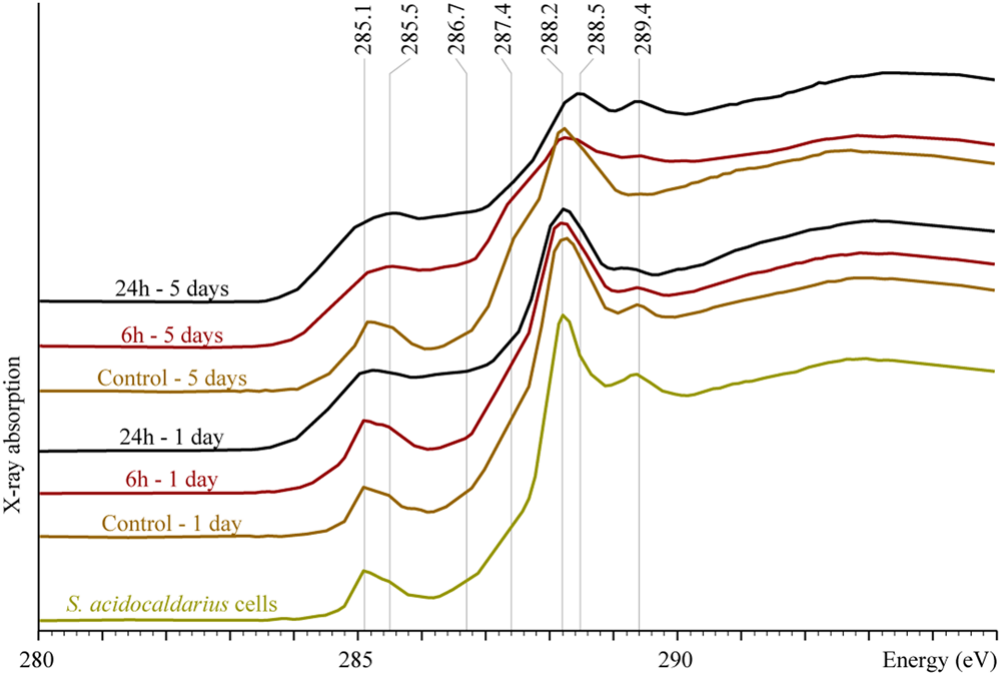
Normalized C K-edge XANES spectra of non mineralized *S. acidolcadarius* cells and cells mineralized for 6 or 24 h and heated for 1 or 5 days at 150 °C.

Before the experiments, cells of *S. acidocaldarius* displayed a C-XANES spectrum typical of bacteria composed of proteins, carbohydrates and lipids (Fig. 4) with a main peak at 288.2 eV, assigned to the 1s → π* electronic transitions in amide groups ((R1,R2)N–C=O), a peak at 289.4 eV corresponding to 1s → 3p/σ* transitions in hydroxyl groups (C-OH), a small peak at 285.1 eV, attributed to 1s → π* transitions in aromatic or olefinic groups (C=C), and a shoulder centered at about 287.3 eV, attributed to 1s → π* transitions in carbonyl (C=O) and phenolic (Ar-OH) groups^14,17,29,53^.

Non-encrusted cells of *S. acidocaldarius* underwent a significant chemical degradation during the 1- and 5-day long experiments at 150 °C. Indeed, the relative concentrations of amide and hydroxyl groups decreased (as evidenced by the intensity decrease of the absorption peaks at 288.2 and 289.4 eV) while the relative concentrations of aromatic/olefinic groups, carbonyl/phenolic groups and carboxyl groups increased (as evidenced by the intensity increase of the absorption features at 285.1, 287.3 and 288.5 eV, respectively). A similar but even more advanced degradation was observed for encrusted cells that had been exposed to the same conditions (Fig. 4).

### Mineralogical evolution upon experimental fossilization

After 1 day at 150 °C, non-encrusted cells of *S. acidocaldarius* evolved into more or less broken spherical particles (Fig. 1) of about 600 nm in diameter (mean 610 +/− 100 nm, n = 40). This spherical morphology was lost after 5 days of experiment and only organic aggregates were recovered (Fig. 1). In contrast, abiotic Fe-phosphates remained spherical during 1 and 5 day long experiments at 150 °C and formed aggregates exhibiting a quite heterogeneous size distribution ranging from 100 nm to 1.5 µm in diameter (530 +/− 310 nm, n = 74) (Fig. 2). Of note, despite thermal treatment, abiotic Fe-phosphates remained amorphous (Fig. 3). This was not the case of the Fe-phosphates that encrusted *S. acidocaldarius* cells: these minerals evolved into crystalline lipscombite (Fe^II^_x_Fe^III^_3−x_(PO_4_)_2_(OH)_3−x_) in all experiments (Fig. 3). They systematically consisted in large crystals as observed by SEM, but morphologies appeared controlled by the duration of the encrustation stage (Fig. 1).

Fe-phosphates encrusting the cells that had been immersed into the mineralization medium for 6 h became “framboids” of lipscombite upon maturation at 150 °C for 1 day, *i.e*. an assembly of ovoid units (Fig. 1F,G). After 1 day of experiment, these framboids measured 17 +/− 5 µm (n = 55) in diameter, each unit having a rather narrow size distribution within a single framboid (e.g. 5.8 +/− 0.2 µm) (Fig. 1F). Some of these framboids exhibited pores (around 200 nm in diameter) at their surface (Fig. 1G). In addition, non mineralized spheres of 540 +/− 100 nm in diameter (n = 115) persisted (Fig. 1G). Similar morphologies could be observed in residues of 5-day long experiments performed on cells encrusted for 6 h (Fig. 1H). Holes of 540 +/− 100 nm in diameter (n = 11) were observed at the surface of these minerals as well. Some of them were partly filled with smaller crystals. Of note, lipscombite crystals of the 5-day long experiments were bigger than those of the 1-day long experiments, with sub-units of framboids measuring 4.4 +/− 3.2 µm in diameter (n = 134, min = 370 nm, max = 16.7 µm). In contrast, Fe-phosphates encrusting the cells that had been immersed into the mineralization medium for 24 h became lipscombite minerals displaying a flattened bi-pyramidal morphology more or less covered by patches of organic matter (Fig. 1J, K). The long axis of these minerals measured 23 +/− 5 µm (n = 100). Some of these minerals exhibited pores at their surface (with a diameter < 100 nm) (Fig. 1K). After 5 days, numerous spikes emerging from the surface of these minerals could be observed (Fig. 1L).

**Figure 5:**
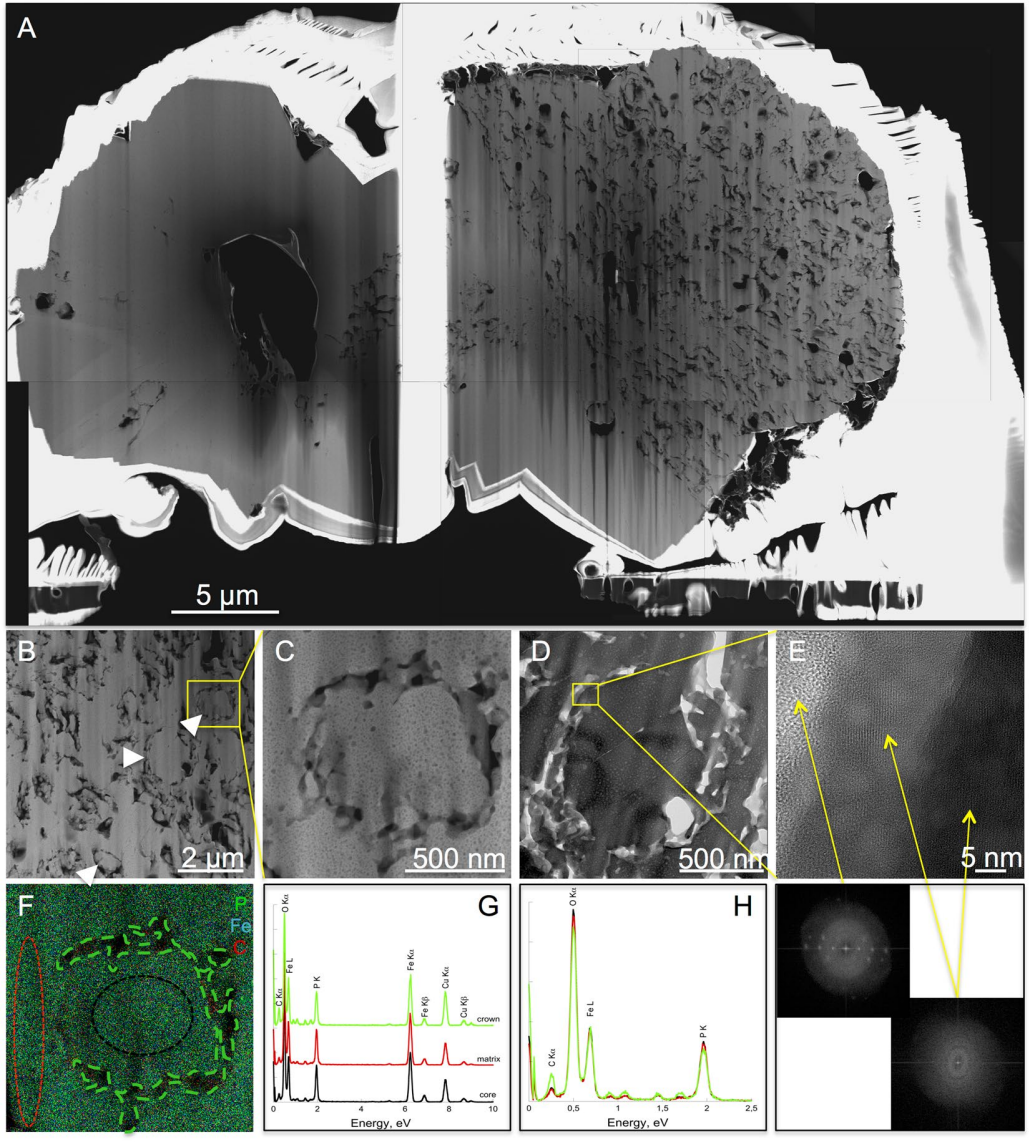
TEM analysis of a FIB section through a framboidal mineral from *S. acidocaldarius* mineralized for 6 h and heated for 1 day at 150 °C in close system. STEM (**A**) and TEM (**B–E**) images of the section. (**E**) close up of the carbon-rich area / mineral interface in (**D**), with corresponding FFT, showing that the carbon-rich region is amorphous, whereas the mineral part corresponds to lipscombite <−211> zone axis. (**F**) EDX map of the cell-like structure in (**C**) and corresponding EDX spectra (**G**,**H**). (**H**) overlay of the EDX spectra shown in (**G**), normalized to the Fe L ray. Colours of the spectra correspond to regions of interest of the same colour in (**F**). Arrowheads point out cell-like structures.

**Figure 6:**
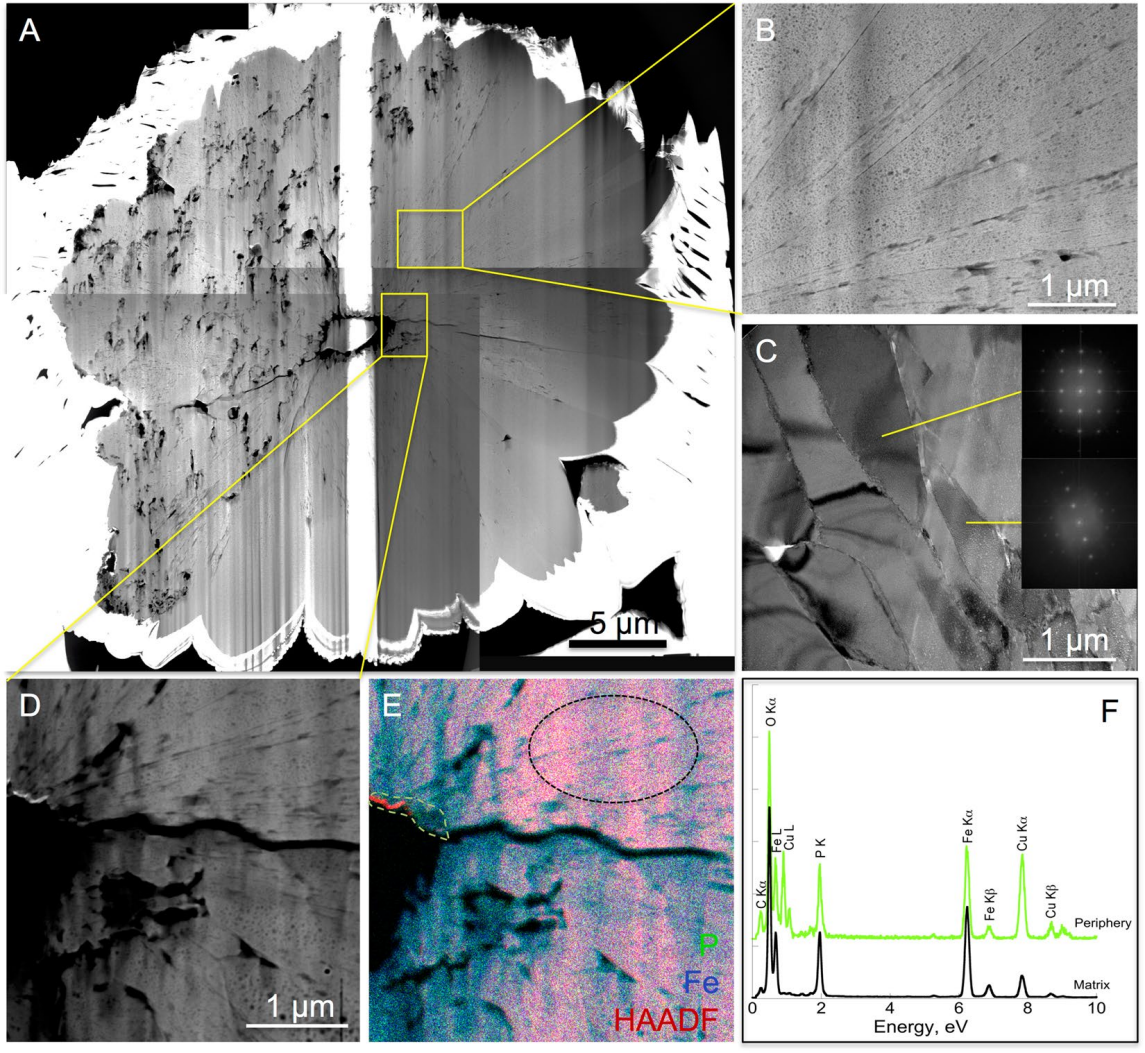
TEM analysis of a FIB section through a framboidal mineral from *S. acidocaldarius* mineralized for 6 h and heated for 5 days at 150 °C in close system. STEM (**A**) and TEM (**B**,**C**) images of the section. FFT corresponding to different domains are consistent with aggregates of lipscombite nanocrystals with different crystallographical orientations. (**D–F**) EDX analysis of the central part of the FIB section (STEM image, EDX map and EDX spectra). Colours of the spectra correspond to regions of interest of the same colour in (**E**).

**Figure 7:**
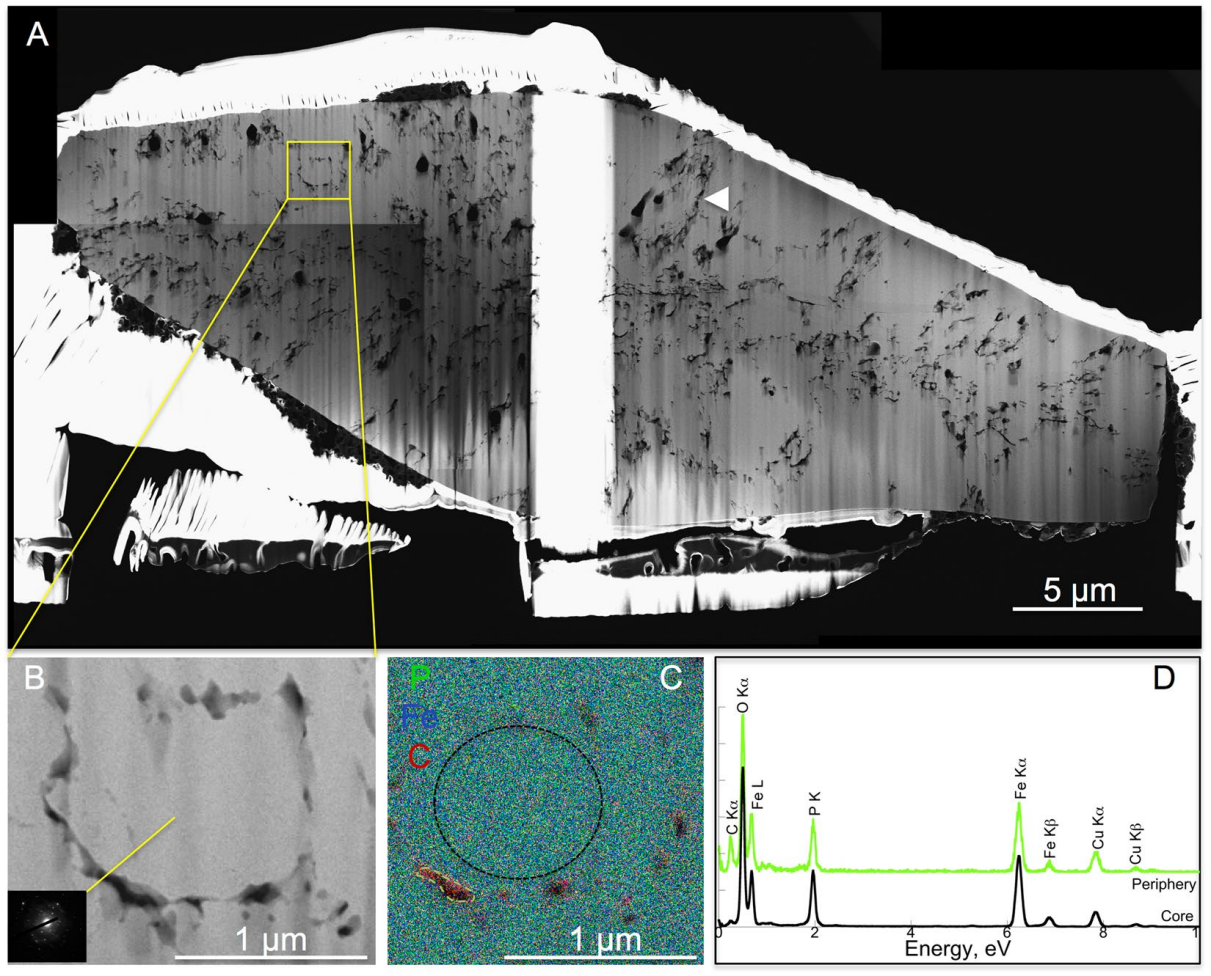
TEM analysis of a FIB section through a bipyramidal mineral from *S. acidocaldarius* mineralized for 24 h and heated for 1 day at 150 °C in close system. STEM (**A**) and TEM (**B**) images of the section and corresponding SAED pattern consistent with lipscombite. (**C**,**D**) EDX map and spectra of the area in (**B**). Colours of the spectra correspond to regions of interest of the same colour in (**C**). Arrowhead in panel A shows a large ovoid possibly resulting from the coalescence of individual cellular residues.

**Figure 8:**
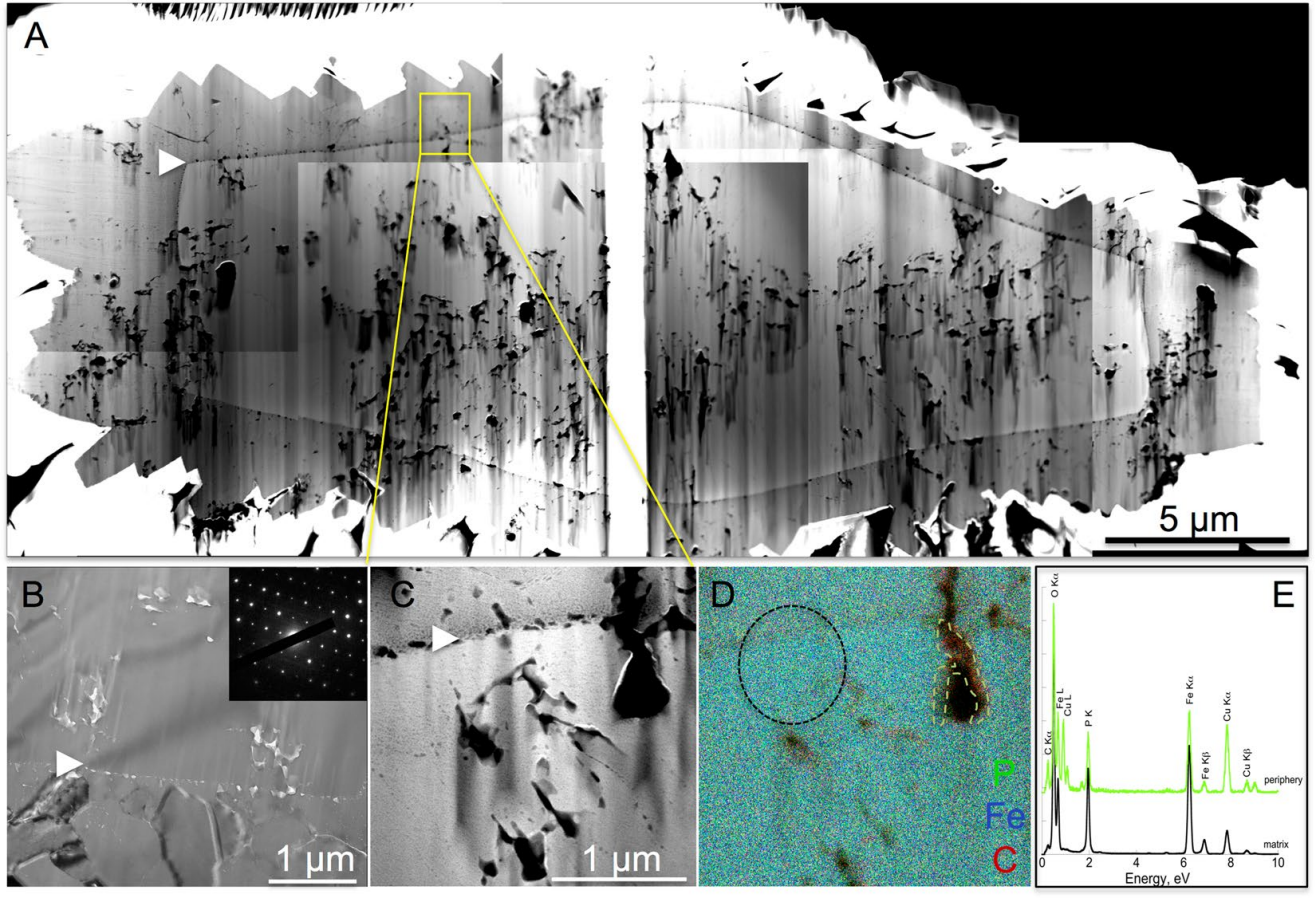
TEM analysis of a FIB section through a bipyramidal mineral from *S. acidocaldarius* mineralized for 24 h and heated for 5 days at 150 °C in close system. STEM (**A**,**C**) and TEM (**B**) images of the section and corresponding SAED pattern consistent with lipscombite (<−110> zone axis). The same SAED pattern is obtained on either sides of the overgrowth boundary, pointed out by arrowheads. (**D,E**) EDX map and spectra of the area in (**C**). Colours of the spectra correspond to regions of interest of the same colour in (**D**).

High resolution TEM (HRTEM) and electronic diffractions (SAED) confirmed that all residues of encrusted cells of *S. acidocaldarius* were composed of crystalline lipscombite (Figs 5D,E, 6C, 7B, 8B). Residues of 5-day long experiments performed on cells encrusted for 6 h exhibited a fibro-radial structure extending from a central part (hole) (Fig. 6A–C). In this FIB-sectioned mineral, some areas consisted in aggregates of micrometre-sized domains with different crystal orientations (Fig. 6C). In all other FIB-sectioned samples (Figs 5, 7–8), the minerals were single crystals of lipscombite. Even the bipyramidal lipscombite with spiky overgrowths (Fig. 8) exhibited the same crystallographic orientation on either sides of the overgrowth boundary (Fig. 8B). Although well crystallized, all observed lipscombites appeared porous with holes or inclusions of amorphous materials quite suggestive of microbial remains (*e.g*. Fig. 5B–E). In particular, cell-like structures (in the form of crowns) with a diameter of around 1.5 µm and a thickness of 61 +/− 24 nm (n = 46) were observed embedded within lipscombite single crystals from the residue of the 1-day long experiment performed on cells encrusted for 6 h (Fig. 5B,C). Such cell-like shapes thus often displayed a diameter and thickness similar to that of the initial cells, although some large ovoids seen in cross section (*e.g*. structure pointed by the white arrow in Fig. 7A) could have formed structures mimicking larger cells possibly through coalescence of cellular residues. EDXS analyses revealed that the amorphous materials included within lipscombite were enriched in C that may correspond to cellular material residues (Figs 5F–H, 6D–F, 7B–D, 8C–E).

## Discussion

### Influence of Fe-phosphates on the thermal maturation of Archaea

The present study provides experimental insights on archaeal burial-induced degradation processes from a molecular point of view. In the absence of Fe-phosphates, the evolution of *S. acidocaldarius* cells with increasing thermal treatment duration is typical of that of organic compounds having been exposed to thermal maturation^14–17,20^, *i.e*. a loss of oxygen-containing functional groups concomitant to an increase of the relative abundance of aromatic structures^54^. In addition, some of the degraded O-bearing functional groups are transformed into carboxyl and/or OH groups. The absence of a sharp 1s → σ* exciton at 291.7 eV, related to the presence of extensive planar domains of highly conjugated aromatic layers^55,56^, indicates that significant pericondensation of aromatic rings did not occur during the experiments.

*S. acidocaldarius* underwent a more advanced degradation in the presence of Fe-phosphates, thereby highlighting that the association with Fe-phosphates is detrimental to the preservation of organic compounds exposed to temperature conditions typical of diagenesis (Fig. 4). A number of experimental studies have highlighted the importance of the close association with minerals (such as quartz, sulfides, carbonates, phosphates, oxides, clays) for the morphological^57–61^ and chemical^17–21^ preservation of soft tissues during burial-induced diagenesis.

To date, experimental studies demonstrated that (bio)encrustation/entombment by/within mineral phases rather limits the degradation of microorganism molecular signatures through specific chemical interactions. For instance, Alléon *et al*.^20^ experimentally evidenced that, because Si-O-C covalent bonds stabilize some organic functional groups, entombment within silica allows the preservation of microorganisms during burial. Li *et al*.^17^ showed that *E. coli* cells encrusted by Ca-phosphate are morphologically and chemically better preserved upon heating than non-encrusted cells. Picard *et al*.^18,19^ pointed out that, when encrusted by Fe-oxyhydroxides, *Acidovorax* sp. BoFeN1 cells and the twisted stalks produced by Fe(II)-oxidizing bacteria can be quite resistant, morphologically and chemically, to diagenetic pressure and temperature conditions.

Here, we show that encrustation by Fe-phosphate rather enhances the chemical degradation of microorganisms during thermal maturation. Because many organic reactions involve changes in the nominal oxidation state of carbon, the presence of minerals that exert an influence on the oxidation state of the system may impact organic thermal maturation^62,63^. Indeed, it has been experimentally shown that the presence of Fe-rich minerals such as hematite and magnetite may promote both the decarboxylation and deamination of amino acids during hydrothermal alteration^62,63^. Here, the presence of Fe-phosphates has thus likely buffered the system similarly to oxidizing conditions, thereby enhancing the degradation of microbial remains likely via organic matter oxidation coupled to Fe-phosphate reduction.

### Lipscombite crystallization upon fossilization of encrusted Archaea

During the present experiments, the Fe-phosphates encrusting archaeal cells exposed to thermal treatments evolved into lipscombite crystals with a morphotype that depended on the duration of the encrustation stage. Natural lipscombite has been reported in pegmatites^64,65^ and in sedimentary concretions in association with other phosphate minerals, such as vivianite, rockbridgeite, fluorapatite or mitridatite^66^. Lipscombite is a solid solution series (Fe^II^_x_Fe^III^_(3−x)_[(OH)_(3−x)_(PO_4_)_2_]) of tetragonal minerals of the lazulite family^67,68^, consisting of chains of face-sharing FeO_6_ octahedra with adjacent iron sites only partially occupied. Preferential crystal growth along the *c* axis has been reported^69^.

Lipscombite may form in aqueous solutions either (a) from dissolved Fe^II^, Fe^III^ and PO_4_^3−^ species (x Fe^2+^ + (3 − x)Fe^3+^ + 2 PO_4_^3−^ + (3 − x) OH^-^ → Fe^II^_x_Fe^III^_(3−x)_((OH)_(3−x)_(PO_4_)_2_))^70^, or (b) from Fe-phosphates exposed to temperatures above 100 °C in the presence of dissolved Fe^II^ and O_2_ species^71^. This second pathway involves the formation and the subsequent oxidation of vivianite (Fe^II^_3_(PO_4_)_2_.8H_2_O) and likely occurred during the present experiments. Initial Fe-phosphates likely partially dissolved and Fe^III^ was likely partially reduced upon oxidation of archeal organic matter at 150 °C, thus providing dissolved Fe^II^ for the precipitation of lipscombite^22,72^.

While they evolved into lipscombite in the presence of archaea, Fe-phosphates remained amorphous in their absence, even after 5 days at 150 °C (Fig. 3), suggesting that microbial organic matter somehow influences lipscombite formation. This was confirmed by the persistence of organic carbon (with morphologies more or less reminiscent of microbial origin) within lispcombite minerals, suggesting that microbial organic matter was strongly involved in the nucleation of these crystals (Figs 5–8). Indeed, microbial organic compounds, including archaeal surface layers, are known to provide cation binding sites for metals such as Fe^33,43^.

The reported typical habitus of synthetic or natural lipscombite is either tetrahedral (bi)pyramid or octahedral “jackstones”^69,70^. In contrast, the lipscombite crystals that formed during the present experiments exhibited specific morphologies that differed according to the level of cell encrustation, *i.e*. to the initial mineral to organic matter ratio. Framboidal lipscombite crystals formed during experiments with slightly encrusted cells (immersion within the mineralization medium for 6 h), leading after 24 h of heating to lipscombite minerals with a pseudo-radial mesostructure, locally composed of an aggregate of micrometre-sized mineral domains. This suggests that a low mineral to organic matter ratio was likely responsible for a large number of nucleation sites, and thus for high nucleation rates, thereby inhibiting the growth of automorph crystals and leading to framboidal morphology, as suggested for framboidal pyrites^73^. Multiple microbial remains became embedded wihtin these lipscombite framboids, some of them being morphologically well preserved. In contrast, flattened and elongated bipyramidal lipscombite single crystals formed during experiments with heavily encrusted cells (immersion within the mineralization medium for 24 h) (Fig. 1). A high mineral to organic matter ratio likely allowed crystal growth to proceed towards automorphous shapes, with a preferential direction of growth along the *c* axis^69^. Single crystals embedded and at the same time filled in the cell-like remnants.

## Concluding Remarks

The present study evidences that Fe-phosphate encrustation of *S. acidocaldarius* cells enhances organic matter degradation during thermal treatment simulating burial-induced diagenesis, likely because of redox reactions. These reactions eventually lead to the precipitation of lipscombite in close association with microbial remains through (1) partial amorphous Fe^III^-phosphate dissolution and subsequent Fe^III^ reduction upon oxidation of organic matter at 150 °C, (2) Fe (re-)binding with organic matter remains, (3) lipscombite nucleation onto cellular material and subsequent crystal growth. In addition to being a potential Eh indicator^66^, lipscombite minerals may thus be seen as proxies of the presence of (biogenic) organic matter prior to diagenesis, their shape informing about the initial mineral to organic matter ratio. Lipscombite could thus constitute a mineralogical biosignature in ancient Fe deposits^72,74^ or in modern Fe-phosphate sedimentary settings (e.g. lake Pavin^39^). Indeed, the present results suggest that cellular microfossils might be encapsulated within Fe-bearing single crystals or clusters of single crystals depending on the initial encrustation stage of the microbial cells. This result might provide insights to interpret the microfossil/pseudofossil record.

Finally, besides the potential applications for the generation of thermostable biocomposites following the extreme biomimetic concepts^75^, the present results may be of interest for biotechnology. Biominerals formed through bacterial Fe biomineralization (and particularly hollow shells) have been shown to provide very efficient electrochemical properties in Li batteries^35,60^. Lipscombite obtained upon the present experimental conditions displayed very specific morphologies, including porous spheres, and it has been shown that lipscombite can incorporate Li ions in its structure and exhibit good electrochemical performances in Li batteries^76,77^. It would thus be worth evaluating the electrochemical performances of the lipscombite minerals formed upon hydrothermal conditions with *S. acidocaldarius* cells.
